# Traditional Chinese biomedical preparation (Huaier Granule) for breast cancer: a PRISMA-compliant meta-analysis

**DOI:** 10.1042/BSR20202509

**Published:** 2020-08-20

**Authors:** Xialei Yao, Wenwen Wu, Kai Qu, Wei Xi

**Affiliations:** 1 Department of Oncology, Liaocheng People’s Hospital, Liaocheng 252000, Shandong Province, China; 2 Department of Hepatobiliary Surgery, The First Affiliated Hospital of Xi’an Jiaotong University, Xi’an 710061, Shaanxi Province, China

**Keywords:** breast cancer, conventional treatment, Huaier Granule, meta analysis, traditional Chinese biomedical preparation

## Abstract

Huaier Granule, a type of traditional Chinese biomedical preparation (TCBP), is considered to be a promising adjuvant therapy for breast cancer. Although an analysis of the published literature has been performed, the exact effects and safety of Huaier Granule remains controversial. Therefore, a wide-ranging systematic search of electronic databases from which to draw conclusions was performed. Data from 27 trials, including 2562 patients with breast cancer were analyzed. The results indicated that, compared with conventional treatment alone, the combination of conventional treatment and Huaier Granule markedly improved patients’ overall response (*P*=0.02) and quality of life (*P*<0.00001), and significantly prolonged 2-year (*P*=0.02), 3-year (*P*<0.0001) and 5-year (*P*=0.004) overall survival rates, and 1-year (*P*=0.003), 2-year (*P*<0.00001), 3-year (*P*<0.00001) and 5-year (*P*=0.03) disease-free survival. The immune function of patients was also significantly enhanced after combined intervention treatment, indicated by clearly increased percentages of CD3^+^ (*P*=0.05), CD4^+^ (*P*<0.00001) and natural killer cells (*P*<0.0001), and CD4^+^/CD8^+^ ratio (*P*<0.00001). The incidence of myelosuppression (*P*=0.001) and hepatotoxicity (*P*=0.05) was lower in breast cancer patients treated with Huaier Granule, whereas other adverse events did not differ significantly between the two groups (*P*>0.05). In summary, results of this meta-analysis suggest that the combination of conventional treatment and Huaier Granule is more effective for the treatment of breast cancer than conventional treatment alone.

## Introduction

Breast cancer is the second most common cancer and the fourth leading cause of cancer-related deaths worldwide [1,2]. It was estimated that 2,088,849 new cases (11.6% of all sites) of breast cancer and 626,679 (6.6% of all sites) breast cancer-related deaths occurred worldwide in 2018 [1,2]. The etiology of breast cancer is still unclear, with possible factors including high aging, obesity, work pressure, genetic factors and so on [3]. Currently, due to the negligence of women regarding the self-inspection and clinical examination of the breast, early detection of breast cancer remains difficult [3,4]. In patients who have been identified with breast tumor, different strategies of management are used such as hormonal therapy, radiation therapy, surgery and chemotherapy [4–8]. In individuals with distant metastasis, managements are typically aimed at enhancing life quality and survival rate [4–6]. It is known that the above conventional treatment methods often fail to remove the tumor completely [9,10]. In addition, the unpleasant side effects of breast cancer treatment are also one of the most motivating factors to find some alternative methods [9,10].

The use of herbs in treating patients with breast cancer is considered a natural alternative, because some plants may contain properties that naturally have the ability to treat breast cancer [4,10–13]. Huaier (Trametes robiniophila Murr) is a sandy beige mushroom that grows on hard wood trees. It has been widely used in Chinese Medicine for nearly 1600 years [14–18]. Huaier granule, the aqueous product of Huaier extract, is an approved traditional Chinese biomedical preparation (TCBP) by Chinese State Food and Drug Administration (SFDA) to be used alone or combined with other drugs in treatment of various malignant tumors including breast cancer, liver cancer, and gastric cancer [17–21]. Recent studies show that the active ingredient in Huaier extract is a proteoglycan, composed of 41.5% polysaccharides, 12.93% amino acids and 8.72% water [18,22]. Additional sucrose, dextrin and soluble starch with a 2:2:1 ratio makes up the adjuvants in Huaier granule [18]. Previous studies demonstrated that Huaier extract could suppress the progression of tumor cells through multiple pathways [17–19,23]. First, Huaier Granule could suppress cancer cell proliferation by inhibiting cyclin B1 expression, promoting G2/M-phase arrest and modulating the PI3K/AKT signaling pathway [23]. Secondly, it can effectively reverse the multidrug resistance of tumor cells and increase the sensitivity of cancer cells to chemotherapeutic agents [17]. In addition, Huaier Granule also could suppress the proliferation and migration of breast cancer cells through inhibiting lncRNA-H19/miR-675-5p signaling pathway and activation of autophagic cell death [19]. Finally, Huaier granule modulates innate immunity through stimulating cytokine release and generation of reactive oxygen species and nitric oxide [18].

Several clinical studies have suggested that patients with breast cancer may benefit from Huaier granule therapy [17,19]. However, despite intensive studies, the clinical efficacy and safety of the combination of conventional treatment and Huaier Granule has not been systematically evaluated. In the present study, we conducted a meta-analysis to investigate the efficacy and safety of conventional treatment combined with Huaier Granule compared with conventional treatment alone for breast cancer, to provide a scientific reference for the design of future clinical trials.

## Materials and methods

The present meta-analysis was performed in accordance with the Preferred Reporting Items for Systematic Reviews and Meta-Analyses (PRISMA) guidelines [24]. This meta-analysis is a secondary research that based on some previously published data. Therefore, the ethical approval or informed consent was not required in this study.

### Search strategy

Eligible prospective controlled clinical trials were searched from nine electronic databases, including the PubMed, Web of Science, EMBASE, Cochrane Library, Medline, China National Knowledge Infrastructure (CNKI), Chinese Biological Medicine Database (CBM), Chinese Scientific Journal Database (CSJD) and the Wanfang database. Papers in English and Chinese published from January 2000 to April 2020 will be included without any restrictions. The search terms included: “Huaier Granule” or “Huaier aqueous extract” or “Trametes robiniophila Murr” combined with “breast carcinoma” or “breast cancer” or “mammary carcinoma” or “mammary cancer” (Supplementary Table S1).

### Eligibility criteria

#### Inclusion criteria

- Patients must be cytologically or pathologically confirmed as having breast cancer;
- All available randomized controlled trials (RCTs) and high-quality prospective cohort studies investigating patients with breast cancer will be included;
- Studies involving more than 30 breast cancer patients; and
- Studies comparing the clinical outcomes of conventional treatment plus Huaier Granule adjuvant therapy (experimental group) with conventional treatment alone (control group); and conventional treatments including surgical operation, radiation treatment and chemotherapy.

#### Exclusion criteria

Studies involving patients with mixed malignancies, non-controlled clinical trials, literature reviews, meta-analyses, meeting abstracts, case reports, duplicate studies, and those with insufficient available data were excluded.

### Data extraction and management

Data were independently extracted by two investigators (Yao, X.L. and Wu, W.W.) according to the same inclusion and exclusion criteria; disagreements were adjudicated by a third reviewer (Qu, K.).

The following data will be extracted from eligible literatures:

- Study characteristics: name of the first author, year of publication, and sample size of included studies.
- Participant characteristics: tumor stage and age of patients.
- Interventions: intervening methods, and dosage, administration route, cycles and duration of treatment of Huaier Granule.
- Outcome and other data: overall response rate (ORR), disease control rate (DCR), Overall survival (OS), disease-free survival (DFS), Quality of life (QoL), immune indexes [CD3^+^, CD4^+^, CD8^+^, Natural killer cells (NK) percentage, and CD4+/CD8+ cell ratios] and adverse effects, et al.

We will attempt to contact the authors to request the missing or incomplete data. If those relevant data are not acquired, they will be excluded from the analysis.

### Quality assessment

To ensure the quality of the meta-analysis, the quality of the included randomized and nonrandomized controlled trials was evaluated according to the Cochrane Handbook tool [25] and Methodological Index for Nonrandomized Studies (MINRRS, Supplementary Table S2), respectively [26].

### Types of outcome measures

#### Main outcomes

The primary outcomes in present analysis included short-term and long-term clinical efficacy, and adverse effects according to Response Evaluation Criteria in Solid Tumors 1.1 (RECIST Criteria 1.1) [27].

- Short-term clinical efficacy: the short-term tumor response included ORR and DCR. ORR was defined as the sum of complete and partial response rates, and DCR was defined as the sum of complete response, partial response and stable disease rates.
- Long-term clinical efficacy: 1-5 year OS (the time from the date of randomization to death from any cause); 1-5 year DFS, (the time from date of random assignment to date of recurrence or death).
- Adverse events: gastrointestinal adverse effects, myelosuppression, and hepatotoxicity, et al.

#### Secondary outcomes

- QoL: QoL was evaluated using the quality-of-life improved rate (QIR) and Karnofsky score (KPS).
- Immune function indicators: the immune function of breast cancer patients was assessed in terms of CD3^+^, CD4^+^, CD8^+^, NK cells percentage, and CD4+/CD8+ cell ratios.

### Statistical analysis

Stata 14.0 (Stata Corp., College Station, TX, U.S.A.) and Review Manager 5.3 (Nordic Cochran Centre, Copenhagen, Denmark) statistical software were used for statistical analyses. Cochrane’s *Q* test and *I*^2^ statistics were used to assess heterogeneity among the studies [28]. If *P*>0.1 or *I^2^*  <  50%, a fixed effects model was used for the meta-analysis; otherwise, a random effects model was used. The Mantel–Haenszel method will be applied for pooling of dichotomous data and results will be presented as risk ratio (RR) with their 95% confidence intervals (CIs). Inverse variance method will be used for pooling of continuous data and results will be presented as standardized mean difference (SMD) with their 95% CIs. A two-tailed *P*<0.05 was considered statistically significant.

The presence of publication bias was investigated using the funnel plots, Begg’s and Egger’s test if 10 or more studies are included in the meta-analysis [29–31]. If publication bias existed, a trim-and-fill method should be applied to coordinate the estimates from unpublished studies, and the adjusted results were compared with the original pooled RR [32].

Sensitivity analysis was performed to explore an individual study's influence on the pooled results by deleting one single study each time from pooled analysis.

## Results

### Search results

**Figure 1:**
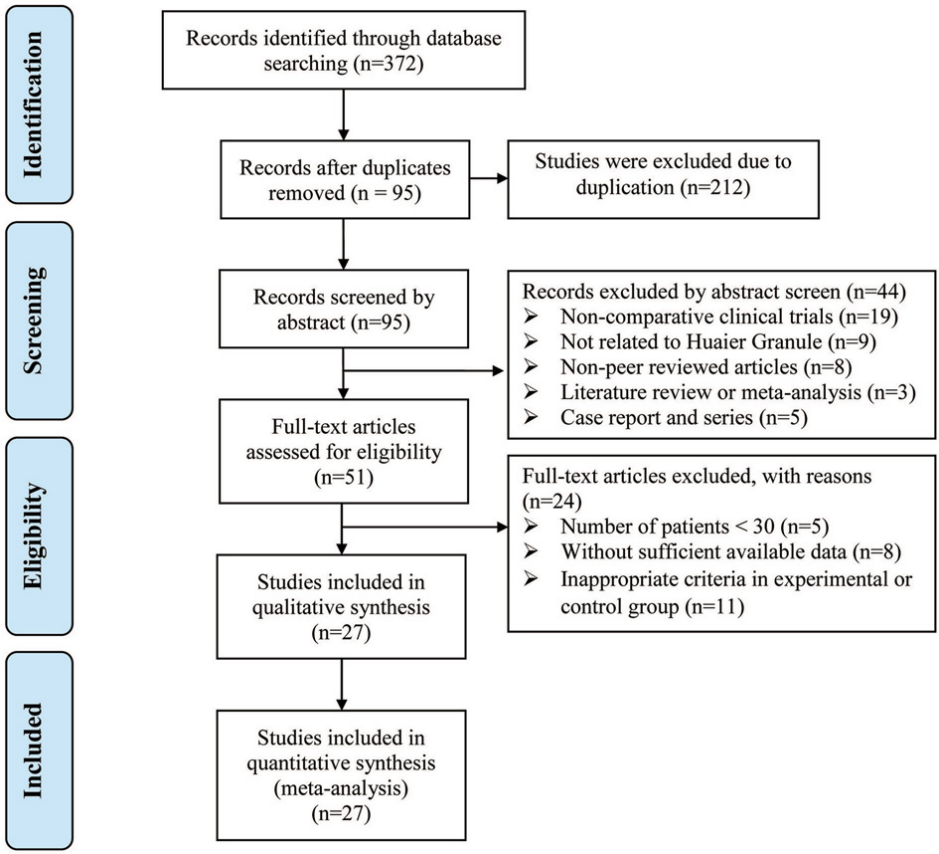
Study selection process for the meta-analysis

The initial search retrieved a total of 372 articles, of which 212 were excluded due to duplication. After title and abstract review, 44 articles were further excluded because they were non-comparative clinical trials (*n*=19), were not related to Huaier Granule (*n*=9), were non-peer reviewed articles (*n*=8), were literature review or meta-analysis (*n*=3), and were case report and series (*n*=5), leaving 51 studies as potentially eligible. After detailed assessment of full texts, studies with <30 breast cancer patients (*n*=5), trials with insufficient data (*n*=8) and inappropriate criteria for the experimental or control groups (*n*=11) were excluded. Ultimately, 27 trials [17,19,33–57], involving 2562 patients with breast cancer, were included in the final analysis (Figure 1).

### Patient characteristics

All included studies were performed in different medical centers in China. In total, 1253 patients with breast cancer were treated using conventional methods in combination with Huaier Granule, while 1309 patients were treated using conventional methods alone. Huaier Granule was manufactured by Qidong Gaitianli Pharmaceutical Co., Ltd, and granted a manufacturing approval number issued by the Chinese SFDA (Z20000109). Study and patient characteristics are summarized in Table 1.

**Table 1:** Clinical information from the eligible trials in the meta-analysis

Included studies	Tumor stage	Patients Con/Exp	Age (year) Control vs Experimental	Intervening methods	Dosage of Huaier granules	Duration of treatments	Parameter types
Chen QJ 2004	II-III	16/22	34–48 (range), 43 (median)	CT vs CT+Huaier granules (OA)	20g/time, 3 times /day	1 month/course, 2 courses.	①, ③
Chen Y 2020	I-IV	50/50	61.42 ± 5.12 vs 61.50 ± 5.16 (mean)	CT vs CT+Huaier granules (OA)	20g/time, 3 times /day	2 weeks for a course, 3 courses.	①, ③
Dai YG 2007	II-III	34/34	32–54 vs 29–55 (range)	CT vs CT+Huaier granules (OA)	20g/time, 3 times /day	2-3 months	⑤
Guo FD 2014	Not provided	25/25	62.1 ± 1.8 vs 59.2 ± 2.3 (mean)	CT vs CT+Huaier granules (OA)	20g/time, 3 times /day	1 month	②
Han SJ 2017	I-III	33/33	49.3 ± 2.1 vs 48.6 ± 2.3 (mean)	CT vs CT+Huaier granules (OA)	20g/time, 3 times /day	3 weeks for a course, 3 courses.	②, ③, ⑤
Lei SS 2016	I-III	39/56	Not provided	CT vs CT+Huaier granules (OA)	20g/time, 3 times /day	1.5 years	④, ⑤
Liang YQ 2015	IV	50/48	35–69 vs 33–68 (range)	CT vs CT+Huaier granules (OA)	20g/time, 3 times /day	6 months	②, ③, ⑤
Li ZH 2016	I-III	219/139	47.5 ± 9.1 vs 47.2 ± 8.8 (mean)	CT vs CT+Huaier granules (OA)	20g/time, 3 times /day	5 months for a course, 2 courses.	②
Lu MQ2017	I-IV	45/45	49.8 ± 8.4 vs 48.5 ± 11.6 (mean)	CT vs CT+Huaier granules (OA)	20g/time, 3 times /day	3 weeks for a course, 2 courses.	④,⑤
Lu Y 2009	I-IV	15/15	53.2 vs 51.7 (median)	CT vs CT+Huaier granules (OA)	20g/time, 3 times /day	3 weeks for a course, 2 courses.	③, ④, ⑤
Qun SX 2020	Not provided	30/35	Not provided	CT vs CT+Huaier granules (OA)	20g/time, 3 times /day	2 years	④, ⑤
Ren XB 2018	I-III	42/42	54.83 ± 2.44 vs 54.12 ± 2.37 (mean)	CT vs CT+Huaier granules (OA)	20g/time, 3 times /day	3 weeks	①, ②, ④
Shan CY 2018	I-III	46/46	53.54 ± 5.58 vs 53.48 ± 5.62 (mean)	CT vs CT+Huaier granules (OA)	20g/time, 3 times /day	3 weeks for a course, 6 courses.	②,③
Tan ZD 2017	I-III	30/31	52.1 ± 5.7 vs 51.2 ± 6.1 (mean)	CT vs CT+Huaier granules (OA)	20g/time, 3 times /day	Not provided	②, ③, ④
Tang Y 2006	I-III	25/25	Not provided	CT vs CT+Huaier granules (OA)	20g/time, 3 times /day	1 month	⑤
Wang MH 2019	I-III	100/101	Not provided	CT vs CT+Huaier granules (OA)	20g/time, 3 times /day	6-18 months	②
Wang W 2019	I-III	48/48	42.1 ± 4.5 vs 40.9 ± 4.0 (mean)	CT vs CT+Huaier granules (OA)	20g/time, 3 times /day	6 months	③
Wu YB 2009	IV	28/24	41–74 (range), 49 (mean)	CT vs CT+Huaier granules (OA)	20g/time, 3 times /day	3 months	②
Xiong Y 2015	I-III	42/50	19–65 vs 20–67 (range)	CT vs CT+Huaier granules (OA)	20g/time, 3 times /day	3 weeks for a course, 6 courses.	②, ⑤
Xu F 2009	II-III	28/32	29–65 vs 27–64 (range)	CT vs CT+Huaier granules (OA)	20g/time, 3 times /day	Not provided	⑤
Yang Z2017	I-II	30/30	55.3 ± 9.6 vs 54.9 ± 8.9 (mean)	CT vs CT+Huaier granules (OA)	20g/time, 3 times /day	3 weeks for a course, 2 courses.	④, ⑤
Yin X 2013	Not provided	20/20	65 ± 1.5 vs 65.5 ± 1.5 (mean)	CT vs CT+Huaier granules (OA)	20g/time, 3 times /day	1 month	②
Zhang JG 2014	I-III	32/32	27–70 vs 28–72 (range)	CT vs CT+Huaier granules (OA)	20g/time, 3 times /day	6 months	②
Zhang Y 2019	Not provided	144/140	22–77 vs 24–80 (range)	CT vs CT+Huaier granules (OA)	20g/time, 3 times /day	6 months	②
Zhao ZW 2020	III-IV	31/31	42.4 ± 1.6 vs 42.8 ± 1.3 (mean)	CT vs CT+Huaier granules (OA)	20g/time, 3 times /day	3 weeks	①, ⑤
Zhong SW 2003	IV	33/29	41–74(range), 49 (median)	CT vs CT+Huaier granules (OA)	20g/time, 3 times /day	3 months	②
Zhou P 2012	I-III	74/70	65–85 vs 65–79 (range)	CT vs CT+Huaier granules (OA)	20g/time, 3 times /day	6 months	②, ③

**Notes:** Control group: conventional treatments alone group; Experimental group: Conventional treatments and Huaier Granule combined group. ①: Overall response rate and Disease control rate; ②: Overall survival or disease-free survival; ③: adverse events; ④: quality of life; ⑤: Immune function index.

**Abbreviations:** CT: conventional treatments; OA: Oral administration.

### Quality assessment

**Figure 2:**
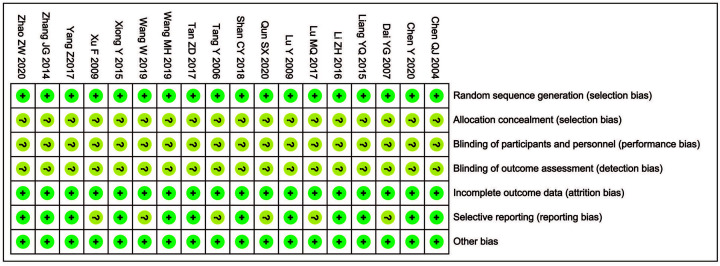
Risk of bias summary Review of authors’ judgments about each risk of bias item for included studies. **Note:** Each color represents a different level of bias: red for high-risk, green for low-risk, and yellow for unclear-risk of bias.

Quality assessment of the risk of bias is shown in Figure 2 and Table 2. The results revealed that the literature retrieved for the present study was of medium and high quality.

**Table 2:** Quality assessment of non-randomized comparative studies

Study	Non-randomized studies								Additional criteria in comparative study				Total
	A	B	C	D	E	F	G	H	I	J	K	L	
Guo FD 2014	2	1	2	1	1	1	2	0	1	2	2	2	17
Han SJ 2017	2	1	2	2	1	1	2	0	1	2	2	2	18
Lei SS 2016	2	1	2	1	1	1	2	0	1	2	2	2	17
Ren XB 2018	2	1	2	2	1	1	2	0	1	2	2	2	18
Wu YB 2009	2	1	2	1	1	1	2	0	1	2	2	2	17
Yin X 2013	2	1	2	1	1	1	2	0	1	2	2	2	17
Zhang Y 2019	2	1	2	2	1	2	2	0	2	2	2	2	20
Zhong SW 2003	2	1	2	1	1	1	2	0	1	2	2	2	17
Zhou P 2012	2	1	2	2	1	2	2	0	1	2	2	2	19

A: A clearly stated aim; B: Inclusion of consecutive patients; C: Prospective collection of data; D: Endpoints appropriate to the aim of the study; E: Unbiased assessment of the study endpoint; F: Follow-up period appropriate to the aim of the study; G: Loss to follow up less than 5%; H: Prospective calculation of the study size; I: An adequate control group; J: Contemporary groups; K: Baseline equivalence of groups; L: Adequate statistical analyses.

**Notes:** The items are scored 0 (not reported), 1 (reported but inadequate) and 2 (reported and adequate).

### Therapeutic efficacy assessments

#### ORR and DCR

**Figure 3:**
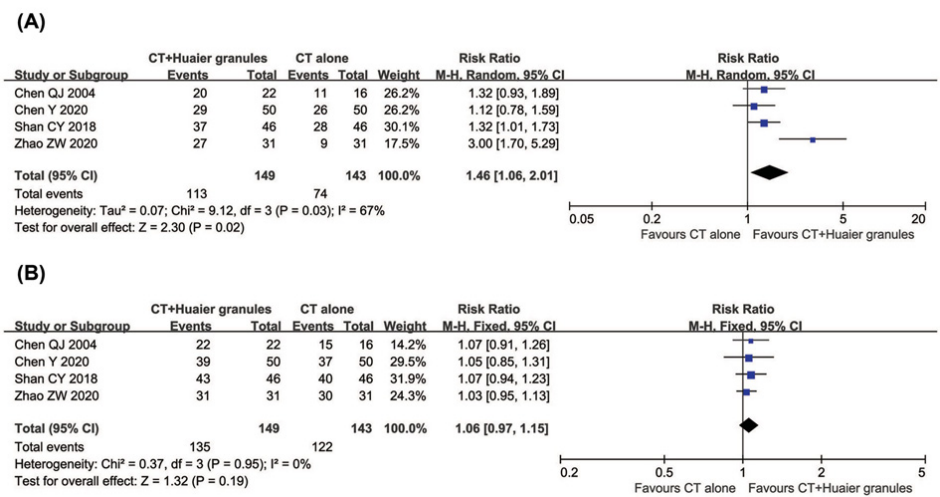
Comparisons of ORR and DCR between experimental and control group Forest plot of the comparison of ORR (**A**) and DCR (**B**) between the experimental and control group. Control group, conventional treatment alone group; Experimental group, conventional treatment and Huaier Granule combined group.

Four clinical trials, involving 292 patients, compared ORR and DCR between the two groups. As shown in Figure 3, the pooled results revealed that patients who underwent combination therapy experienced improved ORR (RR = 1.46, 95% CI = 1.06–2.01, *P*=0.02) and DCR (RR = 1.06, 95% CI = 0.97–1.15, *P*=0.19) compared with those who received conventional treatments alone, although the DCR did not reach significant difference. DCR (*P*=0.95, *I*^2^ = 0%) was not heterogeneous among the studies; therefore, a fixed-effect model was used to analyze RR. Otherwise, a random-effect model was used.

#### Long-term survival

##### 1-year, 2-year, 3-year and 5-year OS

**Figure 4:**
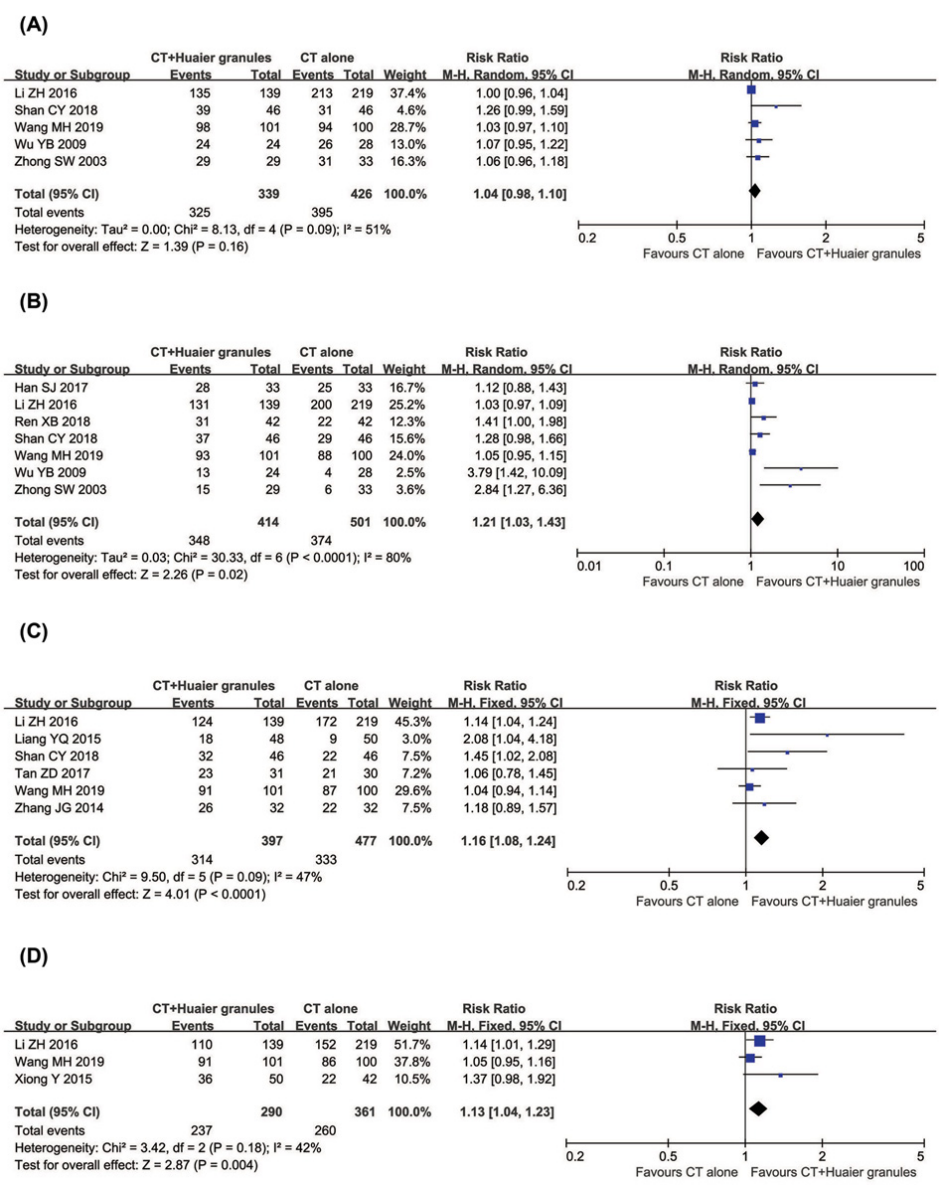
Comparisons of OS between experimental and control group Forest plot of the comparison of 1-year (**A**), 2-year (**B**), 3-year (**C**) and 5-year OS (**D**) between the experimental and control group. Control group, conventional treatment alone group; Experimental group, conventional treatment and Huaier Granule combined group.

Eleven clinical trials with 1,103 breast cancer patients reported OS (Figure 4). Meta-analysis revealed that the 2-year (RR = 1.21, 95% CI = 1.03–1.43, *P*=0.02), 3-year (RR = 1.16, 95% CI = 1.08–1.24, *P*<0.0001) and 5-year OS (RR = 1.13, 95% CI = 1.04–1.23, *P*=0.004) of patients in the combined treatment group were significantly prolonged compared with the control group. There was statistical heterogeneity in 1-year OS (*P*=0.09, *I*^2^ = 51%) and 2-year OS (*P*<0.0001, *I*^2^ = 80%) according to the heterogeneity test. Therefore, a random-effect model was used to pool this meta-analysis. Otherwise, the fixed-effect model was used.

##### 1-year, 2-year, 3-year and 5-year DFS

**Figure 5:**
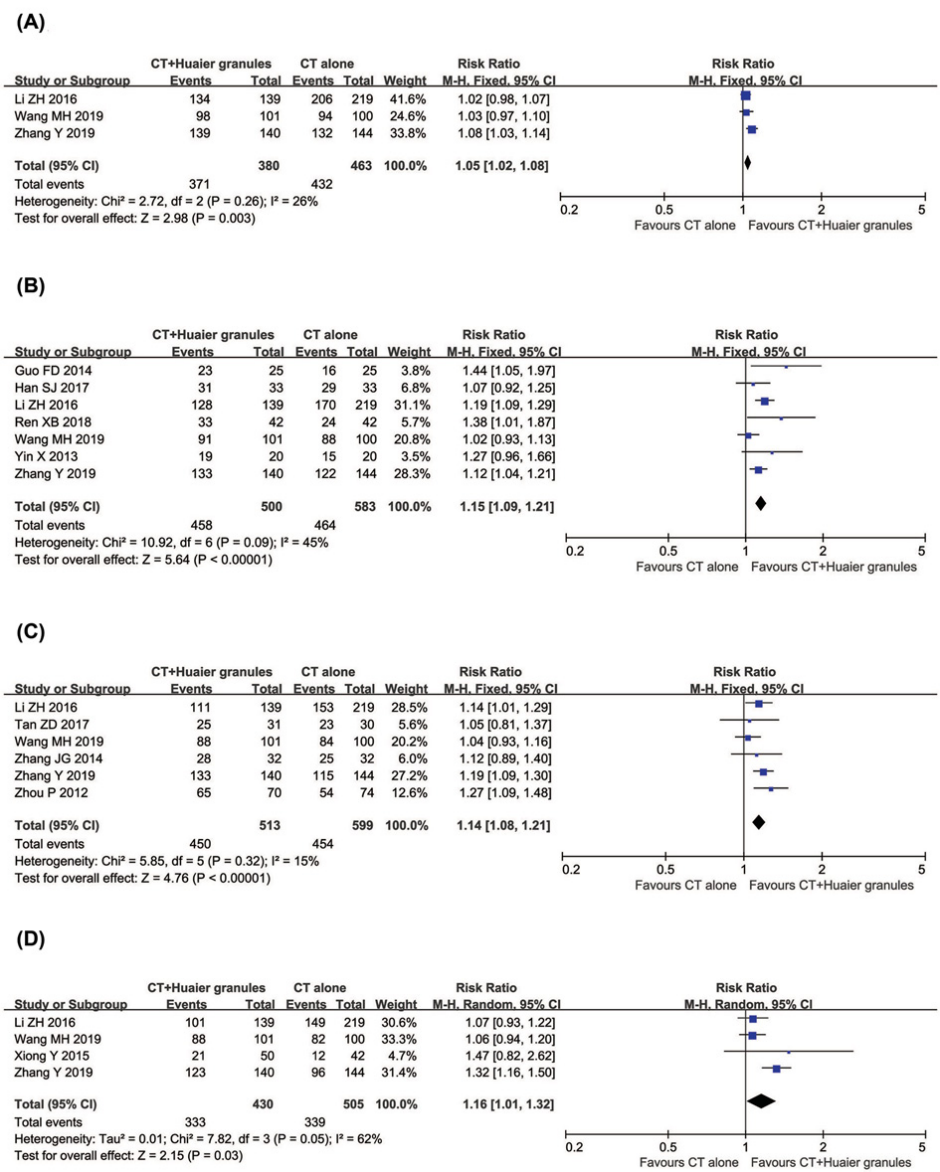
Comparisons of DFS between experimental and control group Forest plot of the comparison of 1-year (**A**), 2-year (**B**), 3-year (**C**) and 5-year DFS (**D**) between the experimental and control group. Control group, conventional treatment alone group; Experimental group, conventional treatment and Huaier Granule combined group.

Ten clinical trials with 1,352 breast cancer patients reported DFS (Figure 5). Meta-analysis revealed that the 1-year (RR = 1.05, 95% CI = 1.02–1.08, *P*=0.003), 2-year (RR = 1.15, 95% CI = 1.09–1.21, *P*<0.00001), 3-year (RR = 1.14, 95% CI = 1.08-1.21, *P*<0.00001) and 5-year DFS (RR = 1.16, 95% CI = 1.01–1.32, *P*=0.03) of patients in the combined treatment group were all significantly prolonged compared with the control group. There was statistical heterogeneity in 5-year DFS (*P*=0.05, *I*^2^ = 62%) according to the heterogeneity test. Therefore, a random effects model was used to pool this meta-analysis. Otherwise, the fixed-effect model was used.

#### QoL assessment

**Figure 6:**
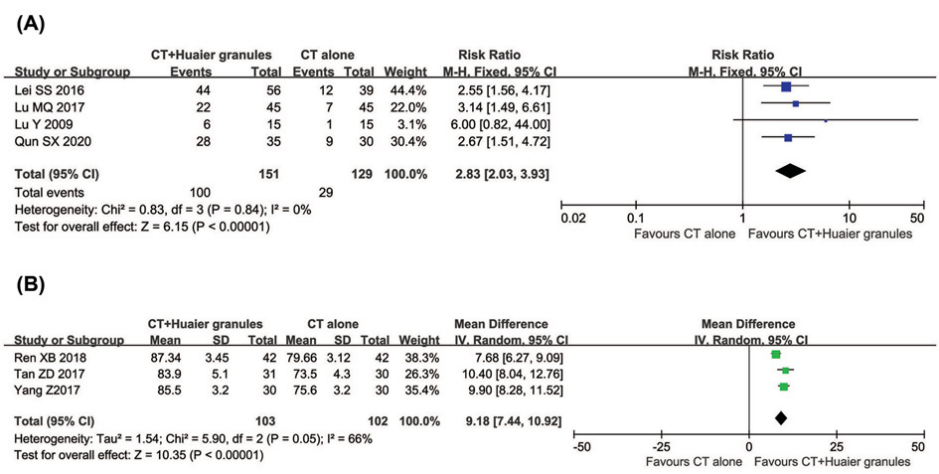
Comparisons of QoL between experimental and control group Forest plot of the comparison of QIR (**A**) and KPS (**B**) between the experimental and control group. Control group, conventional treatment alone group; Experimental group, conventional treatment and Huaier Granule combined group.

Four trials with 280 participants evaluated QIR, and three trials, including 205 patients, reported KPS data (Figure 6). Results demonstrated that the QoL of breast cancer patients in the combined group was significantly better than that of the control group, indicated by significantly increased QIR (RR = 2.83, 95% CI = 2.03–3.93, *P*<0.00001) and KPS (RR = 9.18, 95% CI = 7.44–10.92, *P*<0.00001). QIR (*P*=0.84, *I*^2^ = 0%) was not heterogeneous among the studies; therefore, a fixed-effect model was used to analyze RR. Otherwise, a random-effect model was used.

#### Immune function evaluation

**Figure 7:**
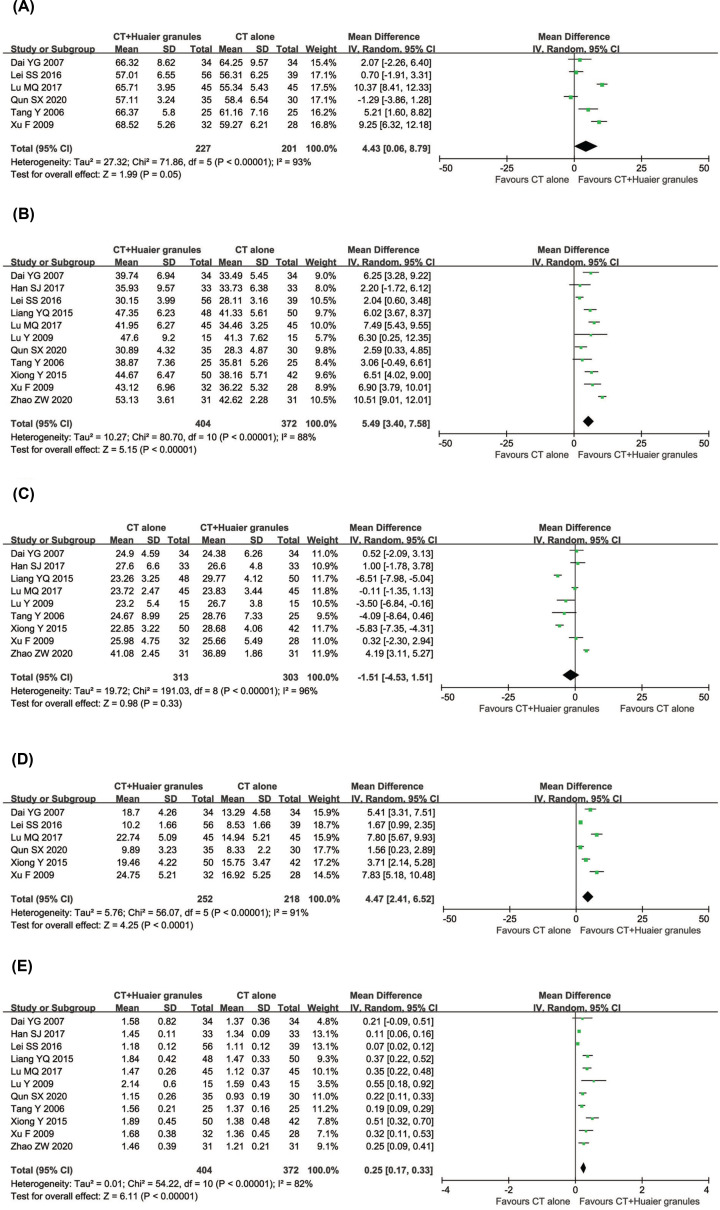
Comparisons of immune function between experimental and control group Forest plot of the comparison of immune function indicators including CD3^+^ (**A**), CD4^+^ (**B**), CD8^+^ (**C**) and NK (**D**) cells percentage and CD4^+^/CD8^+^ ratio (**E**) between the experimental and control group. Control group, conventional treatment alone group; Experimental group, conventional treatment and Huaier Granule combined group; The random effects meta-analysis model (Inverse Variance method) was used.

Immune status of the patients was examined between the two groups in eleven controlled studies including 776 patients (Figure 7). The percentages of CD3^+^ (CD3^+^, RR = 4.43, 95% CI = 0.06–8.79, *P*=0.05), CD4^+^ (RR = 5.49, 95% CI = 3.40–7.58, *P*<0.00001) and NK cells (RR = 4.47, 95% CI = 2.41–6.52, *P*<0.0001), and CD4^+^/CD8^+^ ratio (RR = 0.25, 95% CI = 0.17–0.33, *P*<0.00001) in the combined treatment group were significantly increased compared with those in the conventional treatment alone group, whereas the proportions of CD8^+^ (RR = −1.51, 95% CI = −4.53 to 1.51, *P*=0.33) did not differ significantly between the two groups. A random-effect model was used to pool this meta-analysis due to significant heterogeneity.

#### Assessment of adverse events

**Figure 8:**
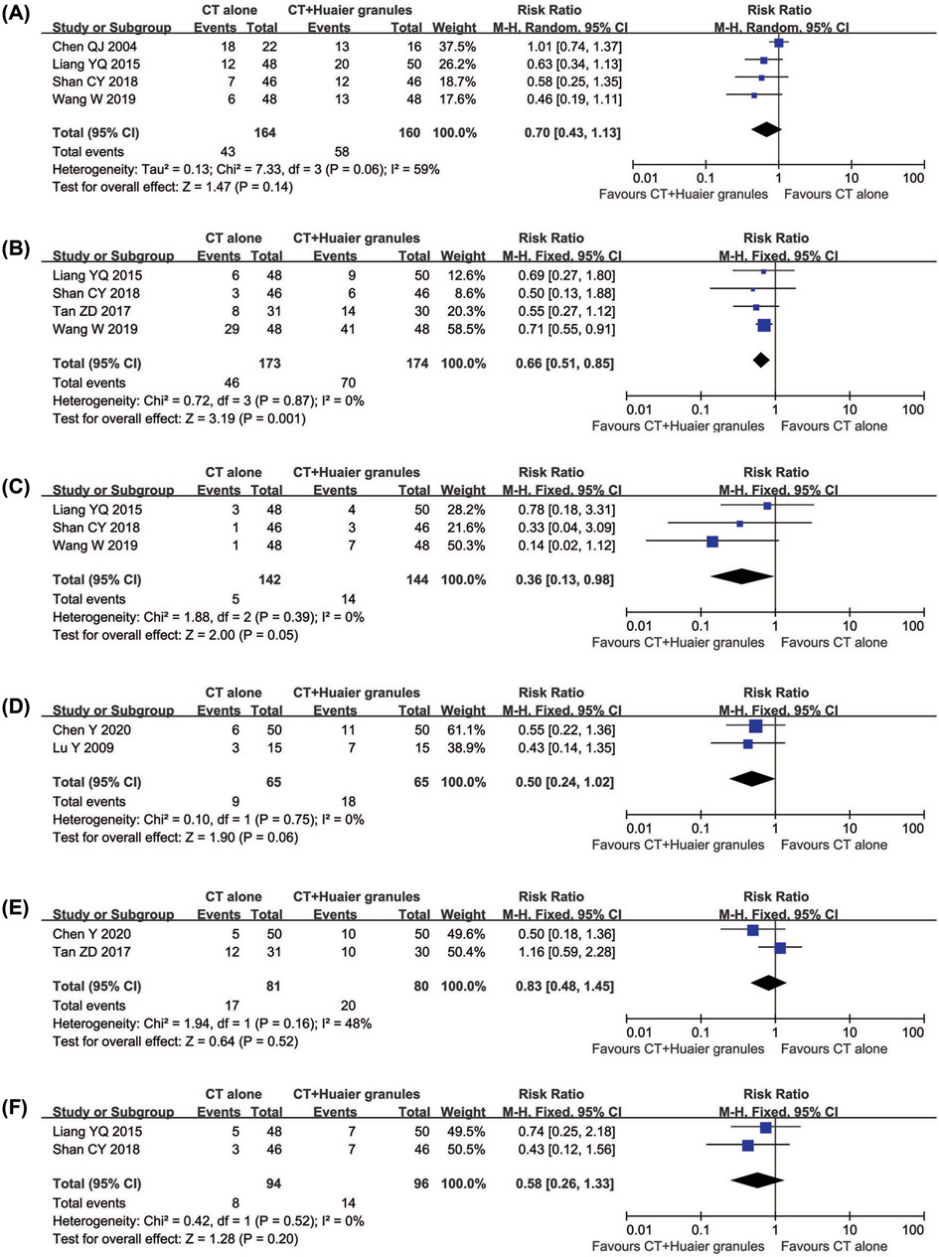
Comparisons of adverse effects between experimental and control group Forest plot of the comparison of adverse effects including gastrointestinal adverse effects (**A**), myelosuppression (**B**), hepatotoxicity (**C**), leukopenia (**D**), nausea and vomiting (**E**) and alopecia (**F**) between the experimental and control group. Control group, conventional treatment alone group; Experimental group, conventional treatment and Huaier Granule combined group.

As shown in Figure 8, patients treated with Huaier Granule and conventional methods exhibited lower incidences of myelosuppression (RR = 0.66, 95% CI = 0.51–0.85, *P*=0.001) and hepatotoxicity (RR = 0.36, 95% CI = 0.13–0.98, *P*=0.05), whereas analysis of gastrointestinal adverse effects (RR = 0.70, 95% CI = 0.43–1.13, *P*=0.14), leukopenia (RR = 0.50, 95% CI = 0.24–1.02, *P*=0.06), nausea and vomiting (RR = 0.83, 95% CI = 0.48–1.45, *P*=0.52), and alopecia (RR = 0.58, 95% CI = 0.26–1.33, *P*=0.20) did not differ significantly between the two groups. There was statistical heterogeneity in gastrointestinal adverse effects (*P*=0.06, *I*^2^ = 59%) according to the heterogeneity test, and a random effects model was used to pool this meta-analysis. Otherwise, the fixed-effect model was used.

#### Publication bias

**Figure 9:**
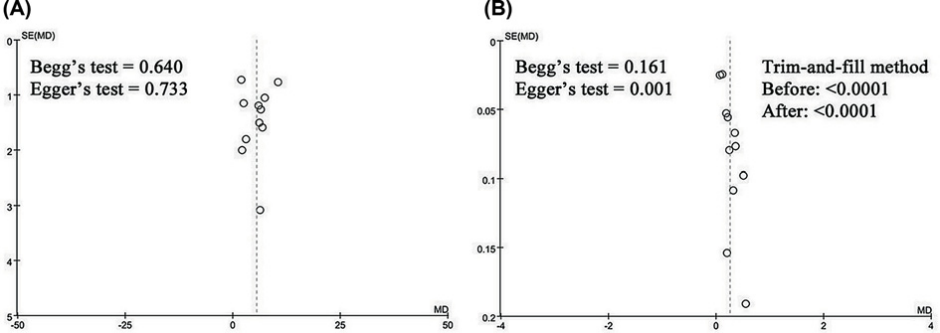
Funnel plot of CD4^+^ (A) and CD4^+^/CD8^+^ (B)

As shown in Figure 9, the funnel plots, Begg’s and Egger’s regression tests results showed that there was publication bias in CD4^+^/CD8^+^ ratio (Begg = 0.161; Egger = 0.001). To determine whether bias affected the pooled risk of CD4^+^/CD8^+^ ratio, a trim-and-fill analysis was performed. The adjusted RR indicated a trend similar to the results of the primary analysis (before: *P*<0.0001, after: *P*<0.0001), reflecting the reliability of the primary conclusions. Parameters discussed less than 10 papers were not conducted publication bias analyses.

#### Sensitivity analysis

**Figure 10:**
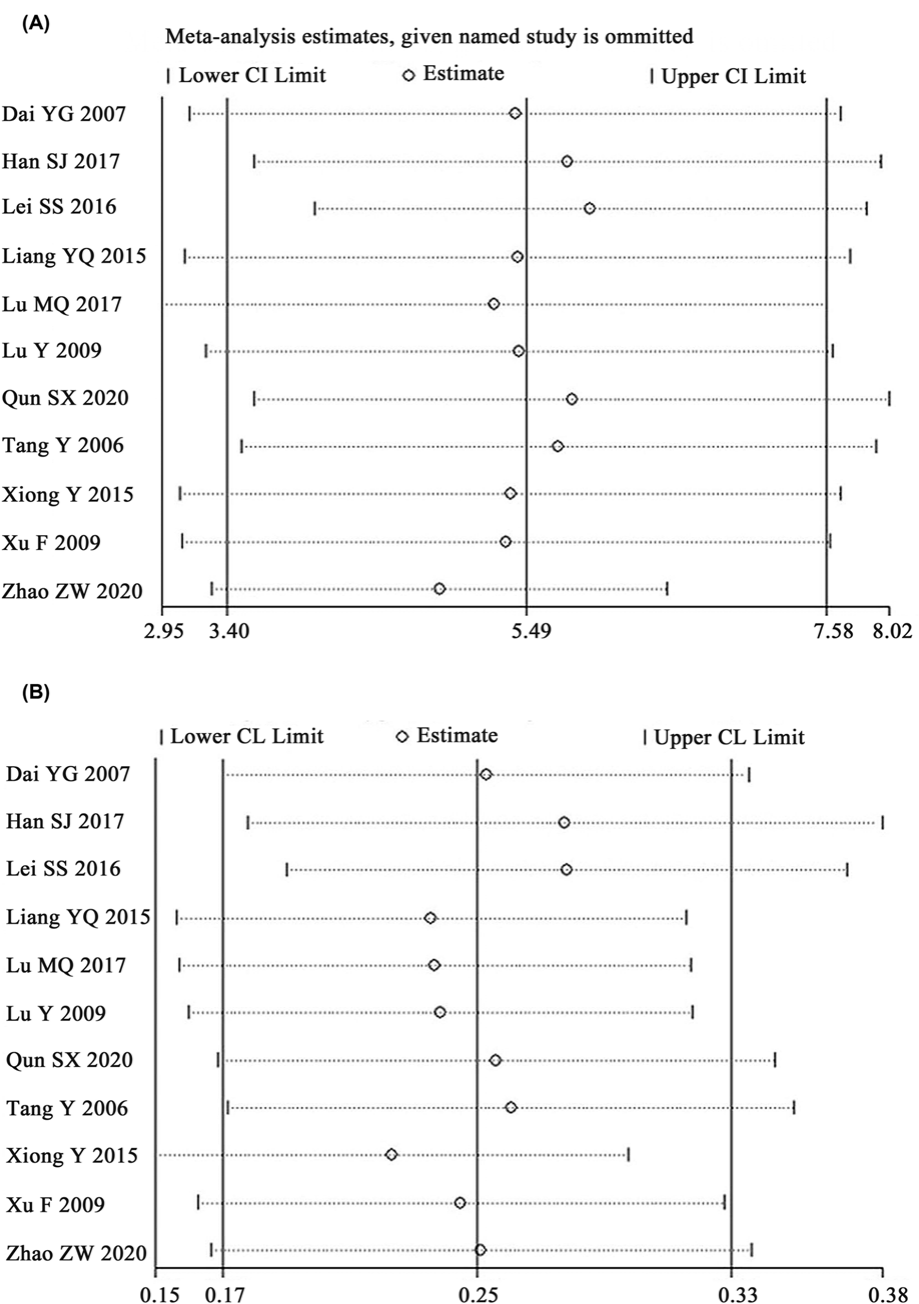
Sensitivity analysis for CD4^+^ (A) and CD4^+^/CD8^+^ (B)

As Figure 10 signified, the results revealed that no individual studies significantly affected the primary indicators (CD4+ and CD4^+^/CD8^+^ ratio), which indicated statistically robust results. Parameters discussed less than 10 papers were not conducted sensitivity analyses.

## Discussion

Huaier granule, the active ingredient of Huaier extract, appears as a light-yellow powder through hot-water extraction, ethanol precipitation, deproteinization and lyophilization procedures[17,18]. As a type of TCBP, Huaier granule has been clinically applied as an effective adjuvant drug in cancer treatment for decades. Although several studies have reported that addition of Huaier Granule could be beneficial to patients with advanced breast cancer [17,19], but the exact therapeutic effects have yet to be systematically evaluated. Thus, in-depth knowledge of the efficacy and safety of Huaier granule is needed. This systematic review will provide a helpful evidence for clinicians to formulate the best postoperative adjuvant treatment strategy for patients with breast cancer, and also provide scientific clues for researchers in this field.

Data from 27 trials [17,19,33–57] including 2,562 patients with breast cancer were included in our meta-analysis. Huaier Granule in all of the included studies was manufactured by Qidong Gaitianli Pharmaceutical Co., Ltd. The dosages of Huaier Granule were 60 g per day via oral administration. The pooled results revealed that the combination of Huaier Granule and conventional treatment for breast cancer achieved more beneficial effects compared with those treated solely with conventional therapy. Compared with conventional treatment alone, Huaier Granule could significantly improve ORR and QoL in patients with breast cancer (*P*<0.05). The study also assessed whether Huaier Granule could prolong the long-term survival rates of breast cancer patients, and the results showed that the 2-, 3- and 5-year OS and 1-, 2-, 3- and 5-year DFS of patients were all significantly prolonged compared with the control group. These results indicated that using Huaier Granule could improve the short- and long-term curative effects of conventional treatment for breast cancer.

T lymphocyte subsets (CD3^+^, CD4^+^, CD8^+^ cell subsets and CD4^+^/CD8^+^ ratio) and NK cells play an important role in antitumor immunity [58]. Studies have shown that patients with advanced cancer showed decreased immune function and NK activity, and exhibiting imbalance of T lymphocytes percentage [58]. Many studies have reported that Huaier Granule can enhance the ability of the body's immunity and resistance to tumors [17,59]. Our analysis demonstrated that the percentages of CD3^+^, CD4^+^ and NK cells, and CD4^+^/CD8^+^ ratio were all significantly increased in breast cancer patients treated with Huaier Granule, indicating that immune function of breast cancer patients was improved after Huaier Granule adjuvant therapy.

Safety is the top priority of clinical treatment. Seven clinical trials with 515 breast cancer patients reported adverse events according to World Health Organization standards. Meta-analysis revealed that patients who underwent Huaier Granule plus conventional treatment demonstrated a lower risk for myelosuppression and hepatotoxicity compared with conventional treatment alone, whereas analysis of other toxic side effects did not differ significantly. Therefore, Huaier Granule appears to be a safe auxiliary anti-tumor medicine for individuals with breast cancer.

There were some limitations to our analysis. Currently, five clinical trials (Table 3) in which breast cancer are being treated by Huaier Granule in conjunction with conventional regimens have been registered on ClinicalTrials.gov (NCT02615457 and NCT02627248) and Chinese Clinical Trial Register (ChiCTR1800015390, ChiCTR-OIC-16007737 and ChiCTR-TRC-11001250). However, except for two studies [17,19], most of the included trials were not registered before the first participant enrolled. Second, as an important Chinese patent medicine, Huaier Granule was mainly applied in China, which may bring an unavoidable regional bias and subsequently influence the clinical application of Huaier Granule worldwide. Third, different trials evaluated the treatment efficacy with different outcomes, resulting in a reduction in the size of the statistical sample, making it difficult to summarize the results at the same scale. Fourth, several results demonstrated significant heterogeneity among the included trials, which may be due to the different tumor stage, tumor subtypes, ages of the breast cancer patients and duration of treatment. However, based on the currently available literature, there are insufficient data to perform more statistical analysis to evaluate correlations. In addition, the efficacy of monotherapy of Huaier Granule in the treatment of breast cancer also needs high-quality evidence to verify. However, up to now, Huaier Granule is mainly combined with radiotherapy, chemotherapy or surgery and other conventional treatment methods for breast cancer. We will keep paying close attention to upcoming high-quality clinical trials in our later studies and carry out further analyses on studies conducted Huaier Granule monotherapy against breast cancer. Finally, publication bias was exists in some indicators, which might because some authors tended to deliver positive results of articles to editors. Therefore, any conclusions need to be made with caution.

**Table 3:** Search results of clinical trial registration

	Registration number	Title	Phase	Conditions	Interventions	Locations
1	NCT02615457	Huaier Granule in Treating Women With Triple Negative Breast Cancer	IV	Triple Negative Breast Cancer	Huaier Granule	Qilu hospital of Shandong University, Ji'nan, Shandong, China
2	NCT02627248	Neoadjuvant Chemotherapy With or Without Huaier Granule in Treating Women With Locally Advanced Breast Cancer That Can Be Removed By Surgery	IV	Breast Cancer	Huaier Granule Other: Chemotherapy	Qilu hospital of Shandong University, Ji'nan, Shandong, China
3	ChiCTR1800015390	Huaier Granule for Stage II and III Triple Negative Breast Cancer with lymph node metastasis: A Multicenter Randomized, Double-blind, Placebo-controlled Clinical Trial	IV	Triple Negative Breast Cancer	Huaier Granule	The First Afliliated Hospital of AMU (Southwest hospital), Chongqing, China
4	ChiCTR-OIC-16007737	A multicenter, double -blind, randomized, placebo-controlled study on stage II-III triple-negative breast cancer with lymph node metastasis treated by Huaier granules	I	Breast cancer	Huaier Granule	Southwest Hospital, The third Military Medical University, Chongqing, China
5	ChiCTR-TRC-11001250	Extract of Fungi of Huaier used for triple negtive breast cancer—a prospective randomized controlled trial	IV	Triple Negative Breast Cancer	Huaier Granule	Southwest Hospital, The third Military Medical University, Chongqing, China

## Conclusion

In summary, findings of this meta-analysis indicate that the combination of Huaier Granule and conventional treatment is effective in treating patients with breast cancer. The clinical application of Huaier Granule not only clearly enhanced the therapeutic effects of conventional treatment, but also effectively improved QoL and immune function in patients with breast cancer. Thus, we anticipate that our study will provide valuable evidence for further evaluation of Huaier Granule. On the other hand, the low quality of some of the included publications increased the risk of bias, which, to some extent, affects the reliability of this research. Therefore, additional studies with high-quality evidence to verify the effectiveness of Huaier Granule-mediated therapy for breast cancer are warranted.

## Supplementary Material

Supplementary Tables S1-S2

## Abbreviations

CBM: Chinese Biological Medicine Database
CI: confidence interval
CNKI: China National Knowledge Infrastructure
CSJD: Chinese Scientific Journal Database
DCR: disease control rate
DFS: disease-free survival
KPS: Karnofsky Performance Score
MINRRS: methodological Index for Non-randomized Studies
NK: natural killer cells
ORR: overall response rate
OS: overall survival
PRISMA: Preferred Reporting Items for Systematic Reviews and Meta-Analyses
QIR: quality-of-life improved rate
QoL: quality of life
RCT: randomized controlled trial
RECIST: Response Evaluation Criteria in Solid Tumors
RR: risk ratio
SFDA: State Food and Drug Administration
SMD: standardized mean difference
TCBP: traditional Chinese biomedical preparation
