# Supplementary material for: Traditional Chinese biomedical preparation (Huaier Granule) for breast cancer: a PRISMA-compliant meta-analysis
Source: Biosci Rep. 2020 Aug 20;40(8):BSR20202509. doi: 10.1042/BSR20202509 (PMC7441486; doi:10.1042/BSR20202509)
Supplement: Supplementary Tables S1-S2 [file BSR-2020-2509_supp.pdf]

Supplement Table 1. Searching strategy for electronic databases

**Data base**	**Search Strategy**
**English database:** PubMed, Web of Science, EMBASE, Cochrane Library and Medline.	**#1.** “breast neoplasm” or “breast carcinoma” or “breast cancer” or “breast tumor” or “breast malignant” or “mammary neoplasm” or “mammary carcinoma” or “mammary tumor” or “mammary cancer” or “mammary malignant” or “BC” or “MC” [Title/Abstract].<br>**#2.** “breast cancer” [MeSH].<br>**#3.** #1 or #2.<br>**#4.** “Huaier” or “Huaier Granule” or “Huaier aqueous extract” or “Trametes robiniophila Murr” or “Trametes robiniophila Murr Granule” or “Trametes robiniophila Murr aqueous extract” [Title/Abstract].<br>**#5. #3** and **#4.**<br>**#6.** limit **#5** to human.<br>**#7.** limit **#6** to (controlled clinical trial).<br>**#8.** limit **#7** to yr=" January 2000-April 2020".

**Chinese database:** China National Knowledge Infrastructure (CNKI), Chinese Biological Medicine Database (CBM), Chinese Scientific Journal Database (CSJD) and the Wanfang database.	**#1.** “ruai” or “ruxianai” or “ruxianzhongwu” or “ruxianzhongkuai” [Title/Keywords].<br>**#2.** “huaier” or “huaierkeli” or “huaiertiquwu” or “huaiershuitiwu” [Title/Keywords].<br>**#3. #1** and **#2**<br>**#4.** limit **#3** to human.<br>**#5.** limit **#4** to (controlled clinical trial)<br>**#6.** limit **#5** to yr="2000.1-2020.4"

PRISMA 2009 Checklist

Section/topic	#	Checklist item	Reported on page #
**TITLE**			
Title	1	Identify the report as a systematic review, meta-analysis, or both.	Page 1 (Title)
**ABSTRACT**			
Structured summary	2	Provide a structured summary including, as applicable: background; objectives; data sources; study eligibility criteria, participants, and interventions; study appraisal and synthesis methods; results; limitations; conclusions and implications of key findings; systematic review registration number.	Page 2 (Abstract)
**INTRODUCTION**			
Rationale	3-4	Describe the rationale for the review in the context of what is already known.	Page 3-4 (Introduction)
Objectives	N/A	Provide an explicit statement of questions being addressed with reference to participants, interventions, comparisons, outcomes, and study design (PICOS).	N/A
**METHODS**			
Protocol and registration	N/A	Indicate if a review protocol exists, if and where it can be accessed (e.g., Web address), and, if available, provide registration information including registration number.	N/A
Eligibility criteria	5-6	Specify study characteristics (e.g., PICOS, length of follow-up) and report characteristics (e.g., years considered, language, publication status) used as criteria for eligibility, giving rationale.	Page 5-6 (Search strategy and selection criteria, Supplementary Table 1)
Information sources	5-6	Describe all information sources (e.g., databases with dates of coverage, contact with study authors to identify additional studies) in the search and date last searched.	Page 5-6 (Search strategy and selection criteria, Supplementary Table 1)
Search	5-6	Present full electronic search strategy for at least one database, including any limits used, such that it could be repeated.	Page 5-6 (Search strategy and selection criteria,

			Supplementary Table 1)
Study selection	6-7	State the process for selecting studies (i.e., screening, eligibility, included in systematic review, and, if applicable, included in the meta-analysis).	Page 6-7 (Data extraction and quality assessment)
Data collection process	6-7	Describe method of data extraction from reports (e.g., piloted forms, independently, in duplicate) and any processes for obtaining and confirming data from investigators.	Page 6-7 (Data extraction and quality assessment)
Data items	7-8	List and define all variables for which data were sought (e.g., PICOS, funding sources) and any assumptions and simplifications made.	Page 7-8 (Outcome definition)
Risk of bias in individual studies	6-7	Describe methods used for assessing risk of bias of individual studies (including specification of whether this was done at the study or outcome level), and how this information is to be used in any data synthesis.	Page 6-7 (Data extraction and quality assessment)
Summary measures	7-8	State the principal summary measures (e.g., risk ratio, difference in means).	Page 7-8 (Outcome definition)
Synthesis of results	8	Describe the methods of handling data and combining results of studies, if done, including measures of consistency (e.g., I^2) for each meta-analysis.	Page 8 (Statistical analysis)

Section/topic	#	Checklist item	Reported on page #
Risk of bias across studies	8	Specify any assessment of risk of bias that may affect the cumulative evidence (e.g., publication bias, selective reporting within studies).	Page 8 (Statistical analysis)
Additional analyses	8	Describe methods of additional analyses (e.g., sensitivity or subgroup analyses, meta-regression), if done, indicating which were pre-specified.	Page 8 (Statistical analysis)
**RESULTS**			

Study selection	9	Give numbers of studies screened, assessed for eligibility, and included in the review, with reasons for exclusions at each stage, ideally with a flow diagram.	Page 9 (Search results, Fig 1)
Study characteristics	9	For each study, present characteristics for which data were extracted (e.g., study size, PICOS, follow-up period) and provide the citations.	Page 9 (Patient characteristics, Table 1)
Risk of bias within studies	9-10	Present data on risk of bias of each study and, if available, any outcome level assessment (see item 12).	Page 9-10 (Quality assessment, Fig 2, Table 2)
Results of individual studies	9	For all outcomes considered (benefits or harms), present, for each study: (a) simple summary data for each intervention group (b) effect estimates and confidence intervals, ideally with a forest plot.	Page 9 (Patient characteristics, Table 1)
Synthesis of results	10-12	Present results of each meta-analysis done, including confidence intervals and measures of consistency.	Page 10-12 (Therapeutic efficacy assessments QoL assessment Immune function evaluation Assessment of adverse events, Fig 3-8)
Risk of bias across studies	12-13	Present results of any assessment of risk of bias across studies (see Item 15).	Page 12-13 (Publication bias, Fig 9)
Additional analysis	13	Give results of additional analyses, if done (e.g., sensitivity or subgroup analyses, meta-regression [see Item 16]).	Page 13 (Sensitivity analysis, Fig 10)
**DISCUSSION**			
Summary of evidence	13-16	Summarize the main findings including the strength of evidence for each main outcome; consider their relevance to key groups (e.g., healthcare providers, users, and policy makers).	Page 13-16 (Discussion)
Limitations	15-16	Discuss limitations at study and outcome level (e.g., risk of bias), and at review-level (e.g., incomplete retrieval of identified research, reporting bias).	Page 15-16 (limitations)
Conclusions	16	Provide a general interpretation of the results in the context of other evidence, and implications for future	Page 16

PRISMA 2009 Checklist

		research.	(Conclusion)
**FUNDING**			
Funding	16	Describe sources of funding for the systematic review and other support (e.g., supply of data); role of funders for the systematic review.	Page 17 (Funding)

From: Moher D, Liberati A, Tetzlaff J, Altman DG, The PRISMA Group (2009). Preferred Reporting Items for Systematic Reviews and Meta-Analyses: The PRISMA Statement. PLoS Med 6(6): e1000097. doi:10.1371/journal.pmed1000097

For more information, visit: **www.prisma-statement.org**.
